# A case of segmental acquired reactive perforating collagenosis: case report and literature review of the unique presentation^☆^

**DOI:** 10.1016/j.abd.2024.03.010

**Published:** 2024-11-09

**Authors:** Yudai Yamauchi, Noritaka Oyama, Minoru Hasegawa

**Affiliations:** Department of Dermatology, Faculty of Medical Sciences, University of Fukui, Fukui, Japan

Dear Editor,

Acquired Reactive Perforating Collagenosis (ARPC) is a difficult-to-treat condition characterized by recurrent, umbilicated papules, the histology of which shows degenerated dermal collagen with subsequent transepidermal elimination.^1^ Diabetes and renal failure can be major precipitating backgrounds,^2^ thus manifesting the widespread eruption distributed in all aspects of the body. We herein report a case of recalcitrant ARCP along the unilateral nerve segment that responded successfully to oral dapsone. We also reviewed 8 similar cases previously reported to discuss the current understanding of this extremely rare clinical presentation.

An otherwise healthy 71-year-old Japanese female presented with a 2-month history of indurative eruptions with a severe itch on the left lumbar area, which have rapidly increased in number. Topical steroids and antihistamines were unhelpful. Examination revealed non-fused, indurative, reddish papular-nodules distributed zonally from the left lower abdomen to the dorsal hip, involving the unilateral nerve segments Th12-L3 (Fig. 1A‒C). The eruptions had well-demarcated central plugs, consistent with dermoscopic findings (Fig. 1D‒E). Tzanck smear test underneath the plugs was negative, and repeated microscopic examination of the crusts was negative for scabies. Laboratory tests, including diabetic markers, and renal and thyroid functions, were all within normal range. Serological tests for varicella zoster and herpes simplex viruses showed a latent infection pattern. Skin light microscopy showed a central erosion filled with a cup-shaped plug containing keratinous debris, collagen fibers, and numerous inflammatory cells mainly consisting of lymphocytes and eosinophils in the dermis (Fig. 2A–B). Vertically oriented collagen fibers, visualized by Elastica van Gieson staining, were extruded from the underlying dermis through the plug (Fig. 2C). There were no skin appendage materials within the plug, estimated by immunostaining using antibodies to pankeratin AE1/AE3 (Fig. 2D). The overall statement suggests the diagnosis of ARPC.

**Fig. 1:**
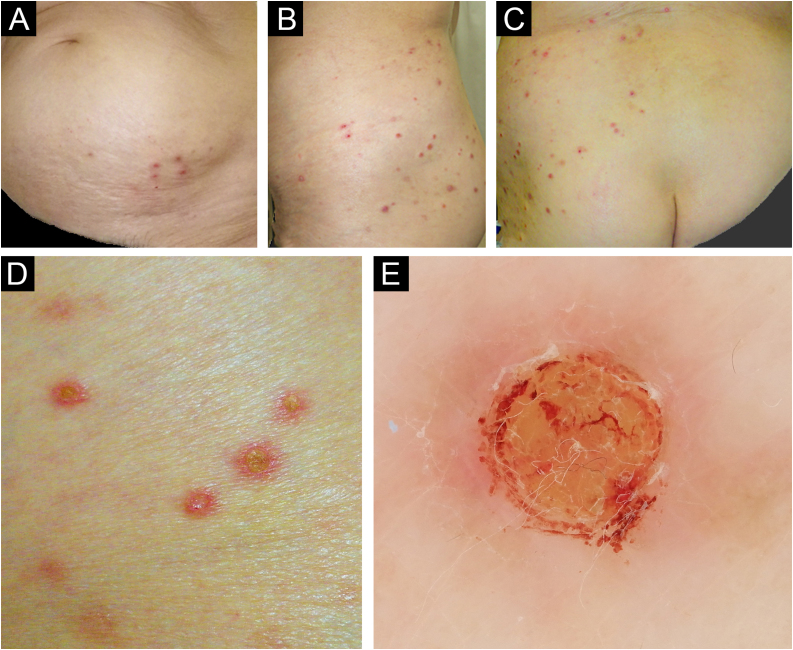
Clinical findings. Clinical pictures showed reddish papules and nodules scattered from the left lower abdomen to the dorsal aspect of the hip, with a zonal arrangement (A‒C). Note that there were no eruptions from the center to the contralateral skin. A close-up view of the skin lesions illustrated non-fused, reddish papulo-nodules with center plugs in each (D), consisting with dermoscopic finding (E).

**Fig. 2:**
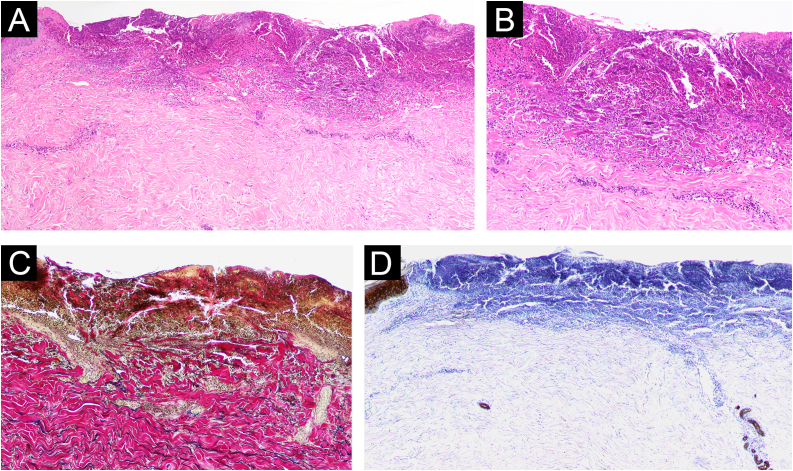
Histological and immunohistological findings. Skin biopsy pathology showed a central erosion filled with a cup-shaped keratinous plug containing numerous inflammatory cells and collagen fibers, and perivascular infiltrates consisting of lymphocytes and eosinophils in the dermis (A, Hematoxylin & eosin, x40; B, Hematoxylin & eosin, x200). Elastica van Gieson staining visualized vertically trans-eliminated collagen fibers from the underlying dermis through the plug (C, ×200). Immunostaining using anti-pankeratin AE1/AE3 antibody revealed no periappendage materials within the plug (D, ×40).

While continuing ultrapotent topical corticosteroid and antihistamine, oral minocycline 200 mg/day was added but was ineffective. We then discontinued minocycline and immediately started oral dapsone 50 mg/day twice daily, which immediately relieved her intractable itching with decreased induration of the eruptions. After 6 weeks, the eruptions flattened and almost disappeared with mild pigmentation (Fig. 3A–B), and did not recur thereafter.

**Fig. 3:**
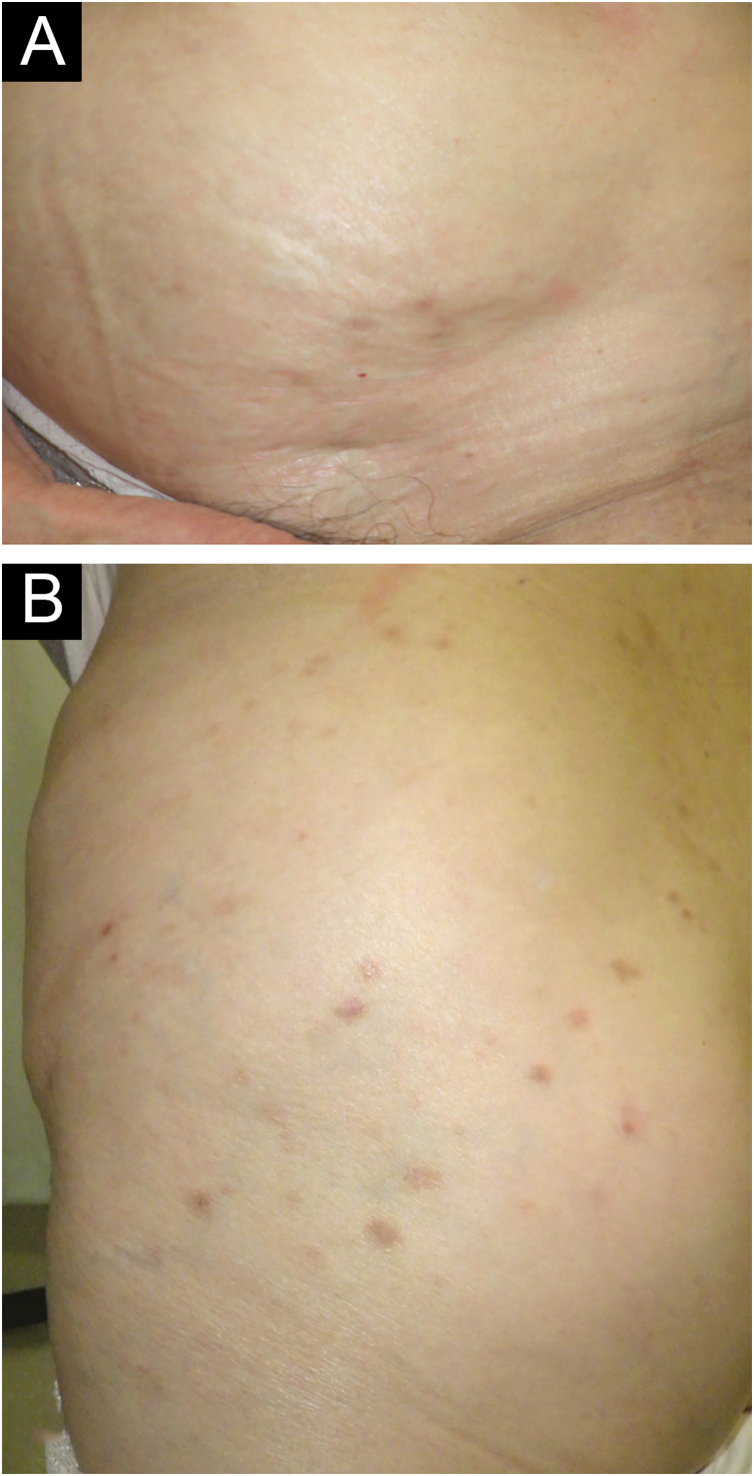
Clinical findings after treatment. After 6 months of oral dapsone treatment, all the eruptions flattened and almost disappeared with mild pigmentation (A, B).

To date, there have been only 9 cases of unilateral ARPC,^2^, ^3^, ^4^, ^5^, ^6^, ^7^, ^8^, ^9^ including our case (Table 1). Because of its unique appearance, it is described as ‘zosteriform’ when the eruption occurs along the unilateral nerve segment.^6^ Their clinical characteristics showed no gender predominance (4 males and 5 females) or site specificity of the affected skin area, although it tended to be more common in middle-aged and elderly individuals (67.0 ± 15.0 years, median ± SD), with an average age of 62.4 years. The treatment and its response varied according to their age and comorbidities. Of the 9 cases, 6 (66.7%) had eruptions within at least 5 months after the development of herpes zoster or even during the treatment course on the same skin sites, implicating a possible involvement of Wolf’s isotopic response seen in some cases with ordinary ARPC and other perforating dermatoses such as Kyrle’s disease. One case had ocurred in the tattooing skin. Interestingly, diabetes and/or chronic renal diseases, both of which are common preceding diseases in ARPC, seem to be less common with a total of 4 cases (44.4%); only 1 had diabetes (1/9, 11.1%), and the remaining 3 had chronic renal failure (3/9, 33.3%).

**Table 1:** Clinical characteristics of unilateral ARPC cases.

Case nº	F/M (y)	Skin lesions	Preexisting herpes zoster	Systemic diseases	Reference nº
1	M (81)	Lt. abdomen (Th10)	5M	NIDDM	3
2	M (63)	Lt. Chest ∼ back	3M	(‒)	4
3	F (67)	Lt. trunk (Th9)	4M	(‒)	5
4	F (66)	Lt. neck, shoulder	2M	(‒)	5
5	M (71)	Rt. arm	1M	CRF	6
6	F (68)	Lt. chest	On-going	CRF	7
7	F (37)	Rt. leg	(‒)	CRF	8
8	M (38)	Lt. forearm	(‒) *Tattooing	(‒)	9
9	F (71)	Lt. abdomen ∼ lumb (Th11-L2)	(‒)	(‒)	Our case

NIDDM, Non-Insuline Dependent Diabetes Mellitus; CRF, Chronic Renal Failure.

Standard treatment strategies for ARPC remain to be established, but appropriate management of associated internal and oncological conditions may help to alleviate the disease activity.^10^ To the best of our knowledge, this case is the first report of unilateral and segmental ARPC without any preexisting medical conditions or even minor trauma, and may therefore provide a different perspective on as yet underrecognized pathogenesis of ARPC, rather than coincidence.

## Authors’ contributions

Yudai Yamauchi: Concepcion; Design; Acquisition of data; drafting the article; analysis of data; final approval of the version to be published.

Noritaka Oyama: Acquisition of data; analysis of data; writing and correcting the article; final approval of the version to be published.

Minoru Hasegawa: Writing and correcting the article; final approval of the version to be published.

## Financial support

None declared.

## Conflicts of interest

None declared.
